# Supplementary material for: Mechanistic insight into the competition between interfacial and bulk reactions in microdroplets through N2O5 ammonolysis and hydrolysis
Source: Nat Commun. 2024 Mar 15;15:2347. doi: 10.1038/s41467-024-46674-1 (PMC10943240; doi:10.1038/s41467-024-46674-1)
Supplement: Supplementary file 1 — Supplementary Information [file 41467_2024_46674_MOESM1_ESM.pdf]

Supplementary Information:

Mechanistic insight into the competition between interfacial and bulk reactions in microdroplets through N_2O_5 ammonolysis and hydrolysis

Ye-Guang Fang[1,2], Bo Tang[1], Chang Yuan[1], Zhengyi Wan[3], Lei Zhao[1], Shuang Zhu[1], Joseph S. Francisco[3*], Chongqin Zhu[1*], Wei-Hai Fang[1]

[1]Key Laboratory of Theoretical and Computational Photochemistry, Ministry of Education, College of Chemistry, Beijing Normal University, Beijing 100875, P. R.

[2]Laboratory of Theoretical and Computational Nanoscience, CAS Key Laboratory of Nanosystem and Hierarchical Fabrication, CAS Center for Excellence in Nanoscience, National Center for Nanoscience and Technology, Beijing 100190, P. R. China

[3]Department of Chemistry, University of Pennsylvania, Philadelphia, Pennsylvania 19104, USA.

[*]To whom correspondence may be addressed.

Joseph S. Francisco, and Chongqin Zhu

Email: frjoseph@sas.upenn.edu or cqzhu@bnu.edu.cn

Supplementary Materials

Section S1. Validation of the SMS-MetaD method

To validate the SMS-MetaD method, we combined it with *ab initio* MD simulations to simulate the hydrolysis of N_2O_5 in bulk water at the revPBE-D3 level of theory. We use the revPBE-D3 here because the neural network potential used in the Galib-Limmer's study[1] was fitted based on the revPBE functional. The bulk system contains one N_2O_5 molecule solvated by 192 water molecules in a 18.06×18.06×18.06 $Å^3$ box with periodic boundary conditions in all three dimensions. **Fig. S1a** shows the collective variable (CV) used in the simulation. **Fig. S1b** displays the free energy profile for the hydrolysis of N_2O_5 as a function of the CV. The free energy barrier for the reaction is 5.8 ± 0.3 kcal/mol, which is in agreement with the free energy barrier obtained by Galib and Limmer using the umbrella sampling method based on the neural network potential (~ 3.8 kcal/mol). In addition, we analyzed the nitrogen-nitrogen distance (R) and the coordination number between the nitrogen atoms in N_2O_5 and the surrounding water molecules (n_w). The results show that the transition state is located at $n_w = 0.5 \pm 0.1$ and $R = 3.15 \pm 0.15$ Å, which is also in agreement with the results of Galib and Limmer ($n_w = 0.4$ and $R = 3.1$ Å). Overall, the SMS-MetaD method and the umbrella sampling method yielded consistent results.

Section S2. Validation of the QM/MM setup

To verify the validity of the QM/MM framework, we combined the SMS-MetaD method with QM/MM MD simulations to study the hydrolysis of N_2O_5 in bulk water. We used the revPBE-D3 to depict the molecules participating in chemical reactions, which contains the N_2O_5 molecule. For the MM model, water molecules were described using the TIP3P model. As shown in **Fig. S2**, the free energy barrier for the reaction is 4.6 ± 0.2 kcal/mol, which is in good agreement with the free energy barrier obtained by Galib and Limmer using a neural network model (~ 3.8 kcal/mol) as well as with our

AIMD simulations (5.8 ± 0.3 kcal/mol). In addition, we analyzed R and n_w in the QM/MM MD simulations. The results show that the transition state is located at n_w = 0.38 ± 0.09 and R = 3.26 ± 0.22 Å, which is also in good agreement with the results of Galib and Limmer (n_w = 0.4 and R = 3.1 Å) as well as our AIMD simulations (n_w = 0.5 ± 0.1 and R = 3.15 ± 0.15 Å).

In addition, we investigated the thermodynamics of N_2O_5 solvation in liquid water using the QM/MM method (revPBE-D3/DZVP//TIP3P). **Fig. S3** depicts the free energy profile for moving a gaseous N_2O_5 into liquid water. The free energy exhibits a global minimum centered at the air-water interface, and this global minimum relative to the gas phase (ΔF_a = -3.5 kcal/mol) corresponds to an interfacial adsorption free energy. Moreover, the difference between the free energy of N_2O_5 in bulk water and that in gas phase (ΔF_s = -1.5 kcal/mol) is defined as the solvation free energy for the gasous N_2O_5. Apparently, the calculated ΔF_a agrees very well with the ΔF_a obtained using the many body potential[2] (ΔF_a = -3.7 kcal/mol), which was parameterized from coupled-cluster calculations, and outperforms that calculated using the neural network potential[1] (ΔF_a = -2.7 kcal/mol). For ΔF_a, although the value calculated by QM/MM is somewhat higher than the one calculated using the many body potential (ΔF_s = -2.7 kcal/mol), it is almost the same as the one calculated based on the neural network potential (ΔF_a = -1.5 kcal/mol). These results validate the effectiveness of the QM/MM method in studying the N_2O_5 reaction.

Supplementary Figures

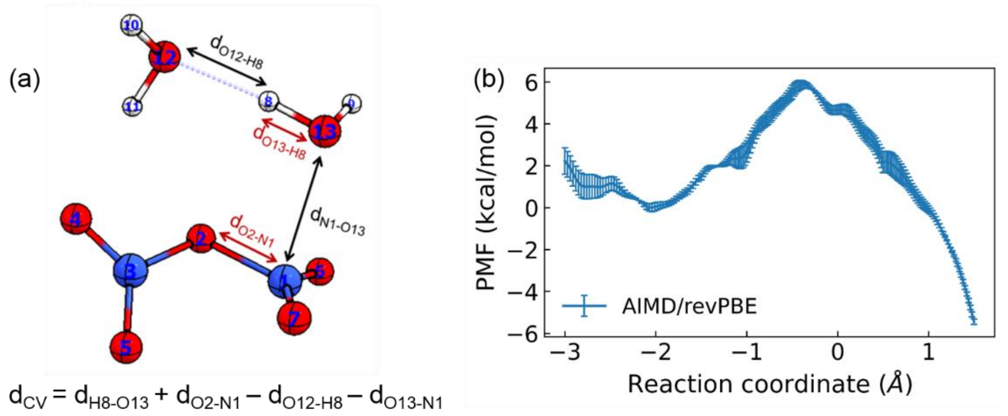

Supplementary Fig. 1. Hydrolysis of N_2O_5 in bulk water from AIMD simulations. (a) Collective variable (CV) used in MetaD-biased AIMD simulation of N_2O_5 hydrolysis in bulk water. The white, red and blue spheres represent hydrogen (H), oxygen (O), and nitrogen (N) atoms, respectively. (b) Free energy profile for N_2O_5 hydrolysis in bulk water via the ionic mechanism simulated at the revPBE-D3 level of theory. The standard deviation of the free energy of the final 8 ps of the MetaD biased QM/MM simulations was used to determine the error bars.

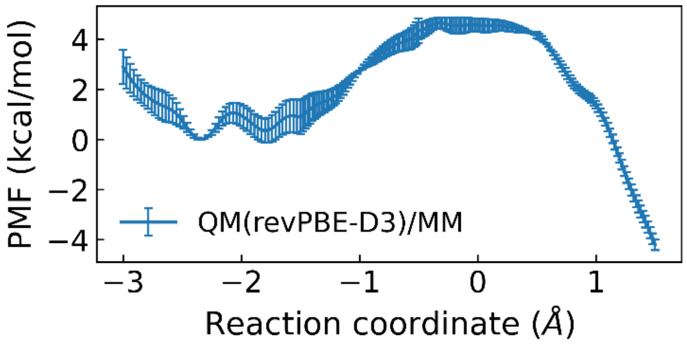

Supplementary Fig. 2. Hydrolysis of N_2O_5 in bulk water from QM/MM MD simulations. Free energy profile for N_2O_5 hydrolysis in bulk water via the ionic mechanism. MetaD-biased QM/MM MD simulation was performed. The QM part was simulated at the revPBE-D3 level of theory. The standard deviation of the free energy of the final 8 ps of the MetaD biased QM/MM simulations was used to determine the error bars.

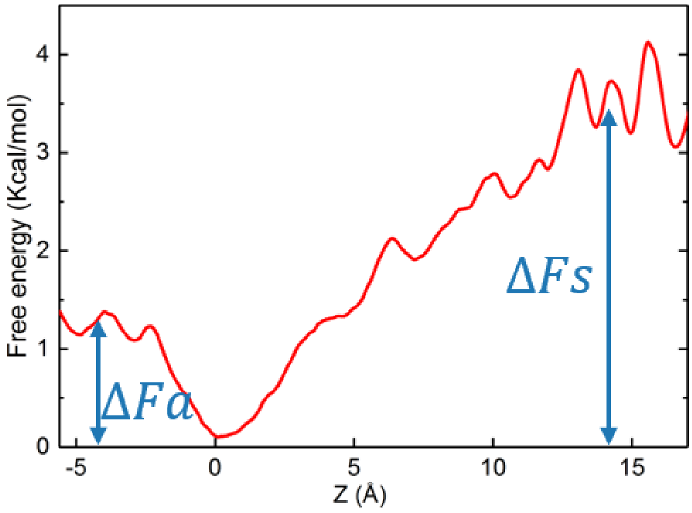

Supplementary Fig. 3. Thermodynamics of N_2O_5 solvation. Free energy profile for moving a gaseous N_2O_5 into liquid water using the QM/MM method (revPBE-D3/DZVP//TIP3P)

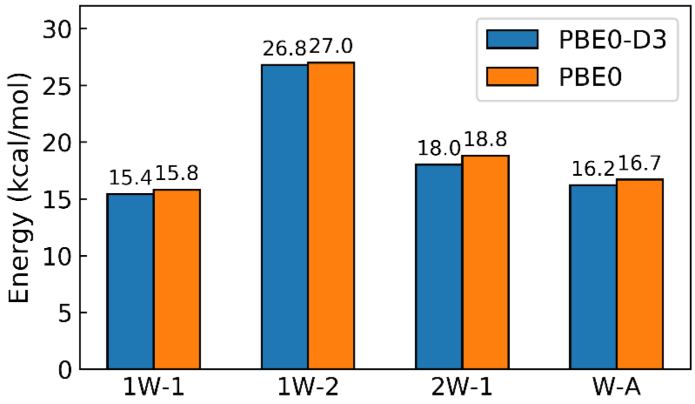

Supplementary Fig. 4. Effect of D3 correction on the reaction barriers. Reaction energy barriers of N_2O_5 hydrolysis for different mechanisms in the gas phase calculated using the PBE0 functional with and without Grimme's D3 correction.

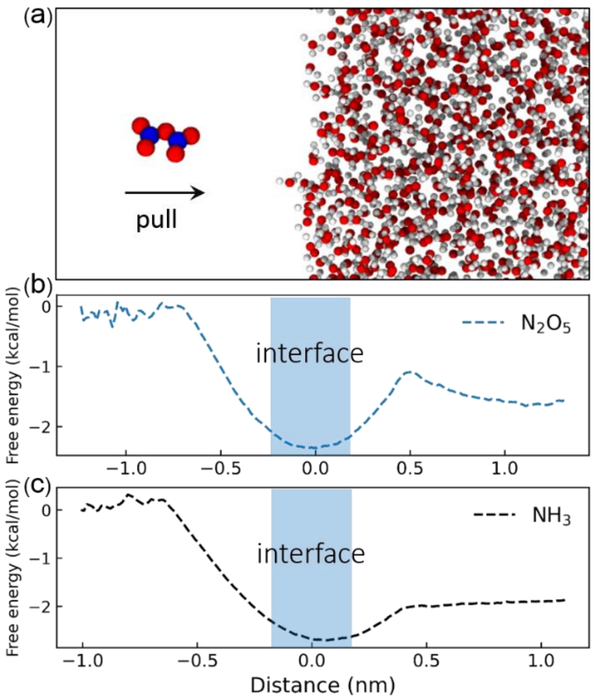

Supplementary Fig. 5. Solvation and adsorption of N_2O_5 and NH_3 in water. **a**, Schematic representation of N_2O_5 near the air-water interface. The white, red and blue spheres represent hydrogen (H), oxygen (O), and nitrogen (N) atoms, respectively. Free energy profiles for the transfer of N_2O_5 (**b**) and NH_3 (**c**) from the gas phase across the air/water interface into the bulk water.

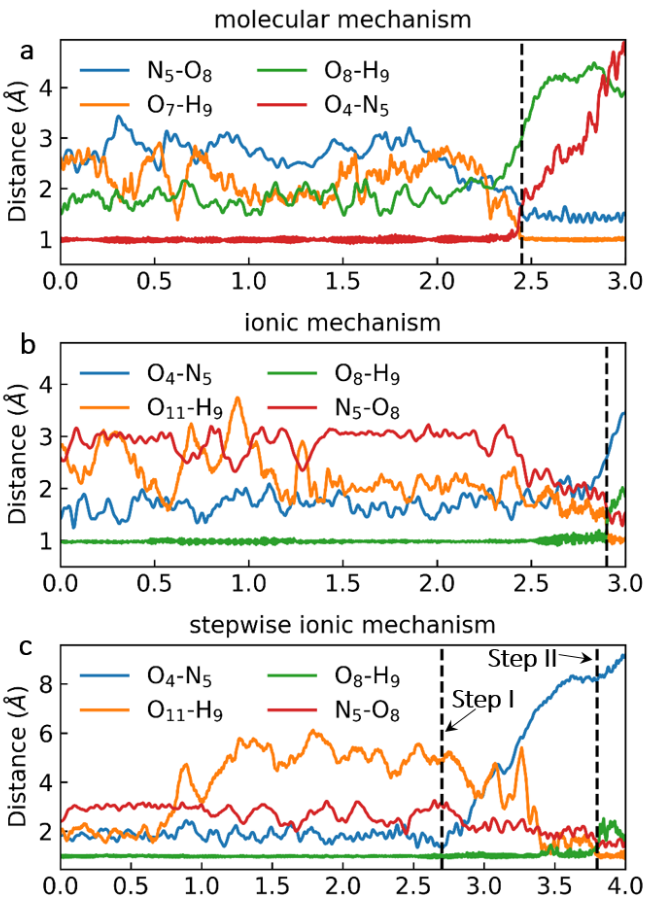

Supplementary Fig. 6. Mechanisms of hydrolysis of N_2O_5 in liquid water. Time evolution traits of key bond distances for N_2O_5 hydrolysis in liquid water via the molecular (a), ionic (b), or stepwise ionic mechanism (c) in MetaD-biased QM/MM MD simulations. Corresponding snapshot structures are shown in Fig. 2 in the main text. The dashed vertical line indicates the moment when a concerted or stepwise reaction is about to occur.

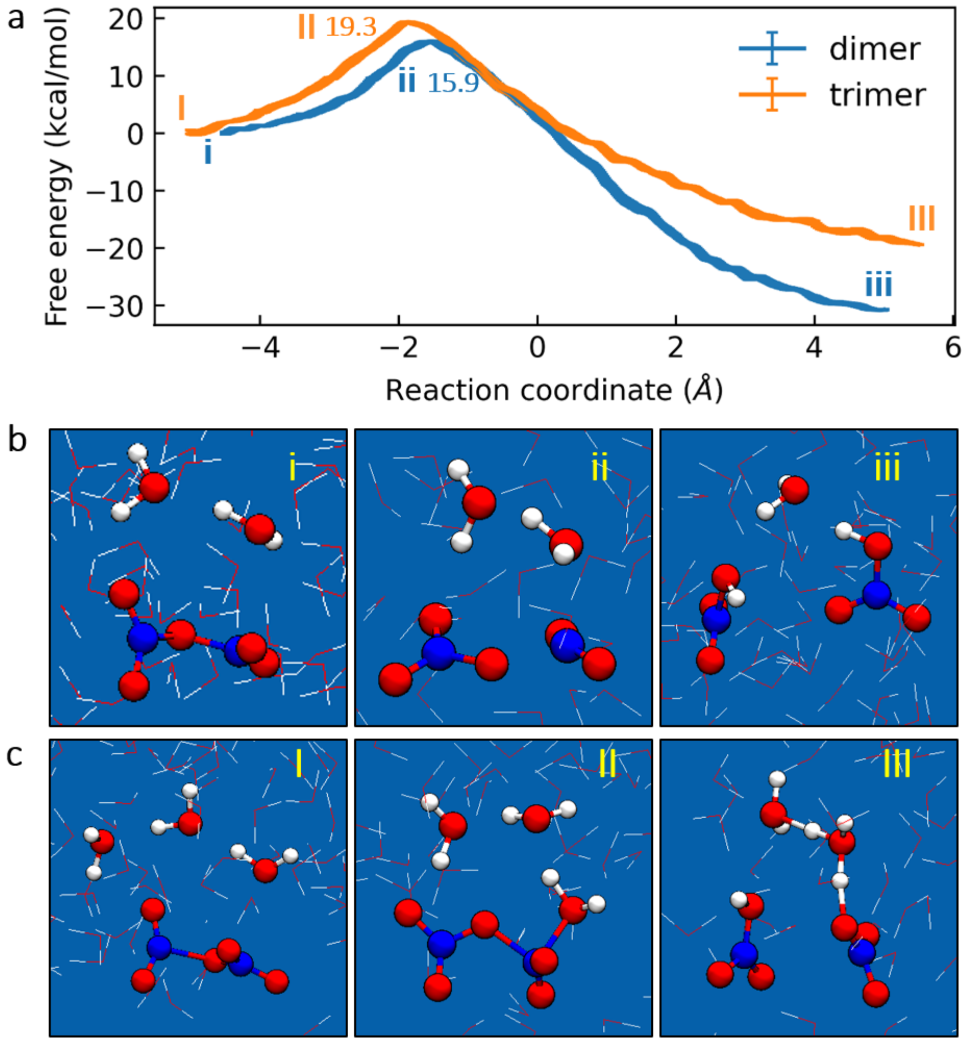

Supplementary Fig. 7. Free energy profiles for N_2O_5 hydrolysis at the air-water interface via the molecular mechanism. a, Free energy profiles for the reaction of N_2O_5 with water dimer (blue line) and trimer (orange line) via the molecular mechanism at the air-water interface. The standard deviation of the free energy of the final 8 ps of the MetaD biased QM/MM simulations was used to determine error bars. Energy barriers are presented in kcal/mol. Snapshot structures of the reaction between N_2O_5 and water dimer (**b**) or water trimer (**c**) captured from MetaD-biased QM/MM MD simulations at the PBE0/DZVP level. The white, red and blue spheres represent hydrogen (H), oxygen (O), and nitrogen (N) atoms, respectively.

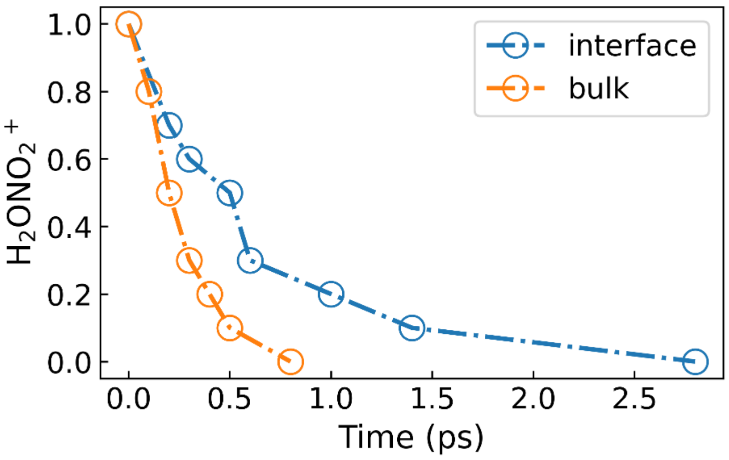

Supplementary Fig. 8. Stability of the intermediate $H_2ONO_2^+$. The fraction of unreacted intermediate $H_2ONO_2^+$ at the air-water interface or in bulk water as a function of simulation time in all independent unbiased QM/MM MD simulations at the PBE0-D3/DZVP level. The average lifetimes of $H_2ONO_2^+$ at the air-water interface and in bulk water are 0.78 and 0.31 ps, respectively.

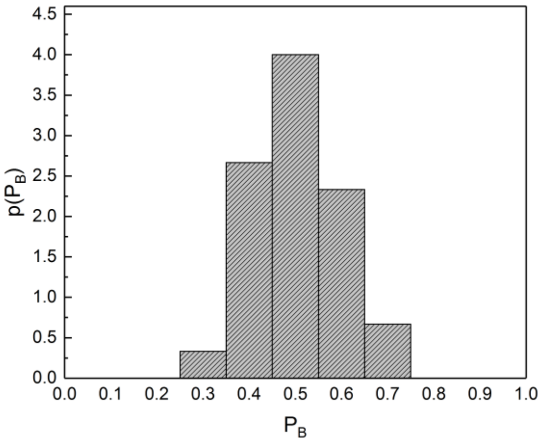

Supplementary Fig. 9. Committor analysis of the reaction coordinate. Distribution $p(P_B)$ of the probability for relaxing to the product within the constrained ensemble with reaction coordinate (RC) ∈ [-0.025, 0.025] for N_2O_5 hydrolysis in bulk water via ionic mechanism.

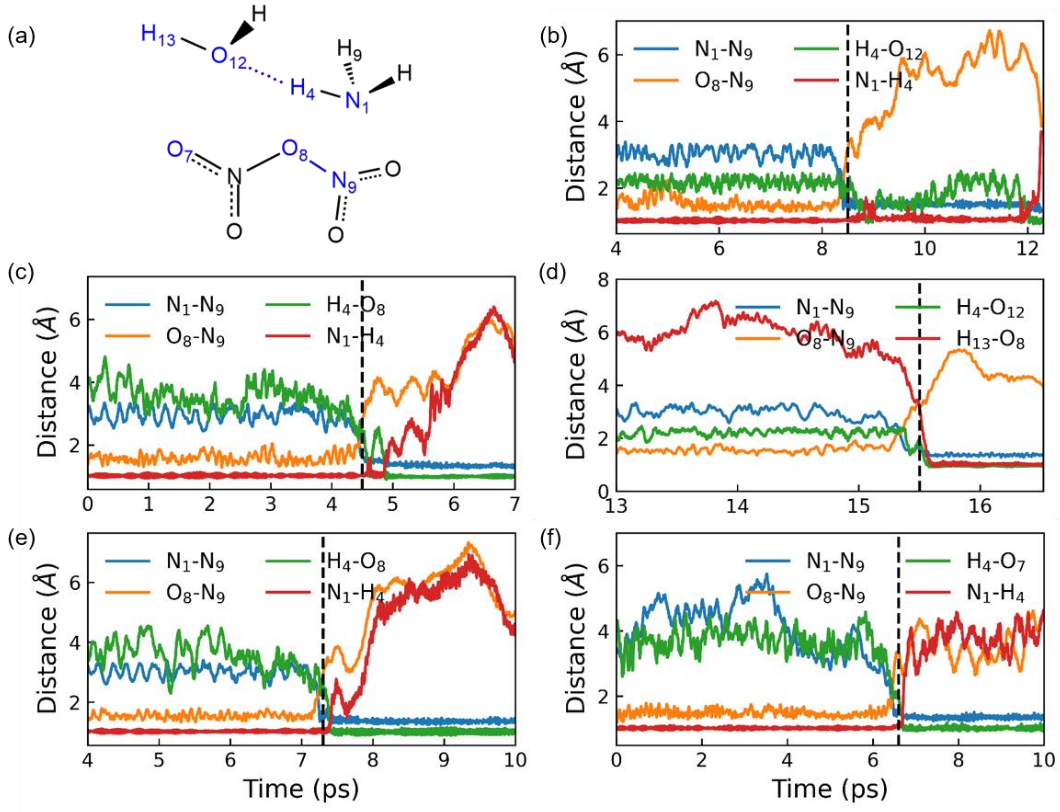

Supplementary Fig. 10. Ammonolysis of N_2O_5 at the air-water interface via molecular mechanism. Time evolution of key bond distances for N_2O_5 ammonolysis at the air-water interface via or molecular mechanism (**b**, **c**, **d**, **e**, **f**). The dashed vertical line indicates the moment when a concerted or stepwise reaction is about to occur. Relevant atomic numbering is depicted in (**a**).

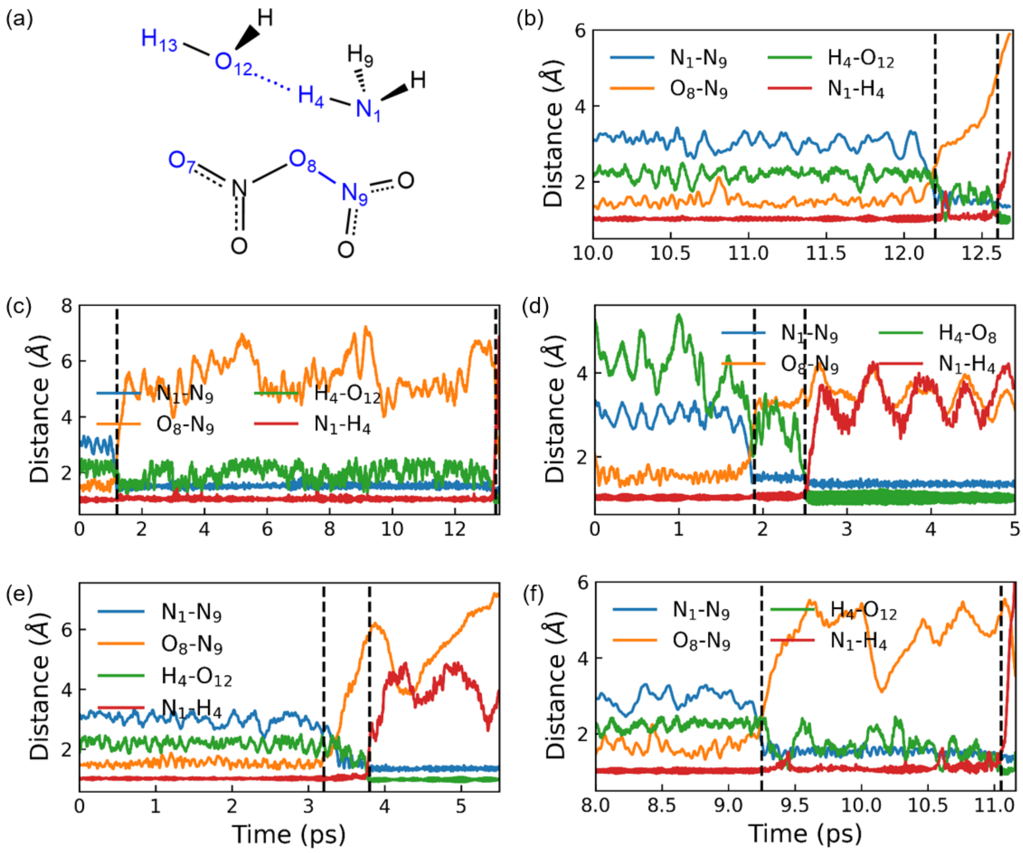

Supplementary Fig. 11. Ammonolysis of N_2O_5 at the air-water interface via stepwise ionic mechanism. Time evolution of key bond distances for N_2O_5 ammonolysis at the air-water interface via or stepwise ionic mechanism (**b**, **c**, **d**, **e**, **f**). The dashed vertical line indicates the moment when a concerted or stepwise reaction is about to occur. Relevant atomic numbering is depicted in (**a**).

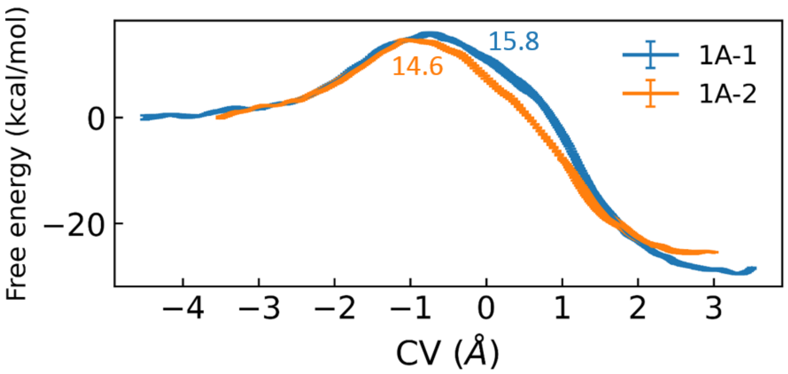

Supplementary Fig. 12. Free energy profiles for N_2O_5 ammonolysis in the gas phase. In the reactions, an H group of NH_3 can attach to the terminal or central oxygen atom of O_2NONO_2. Energy barriers are presented in kcal/mol. The standard deviation of the free energy of the final 8 ps of the MetaD biased QM/MM simulations was used

to determine the error bars.

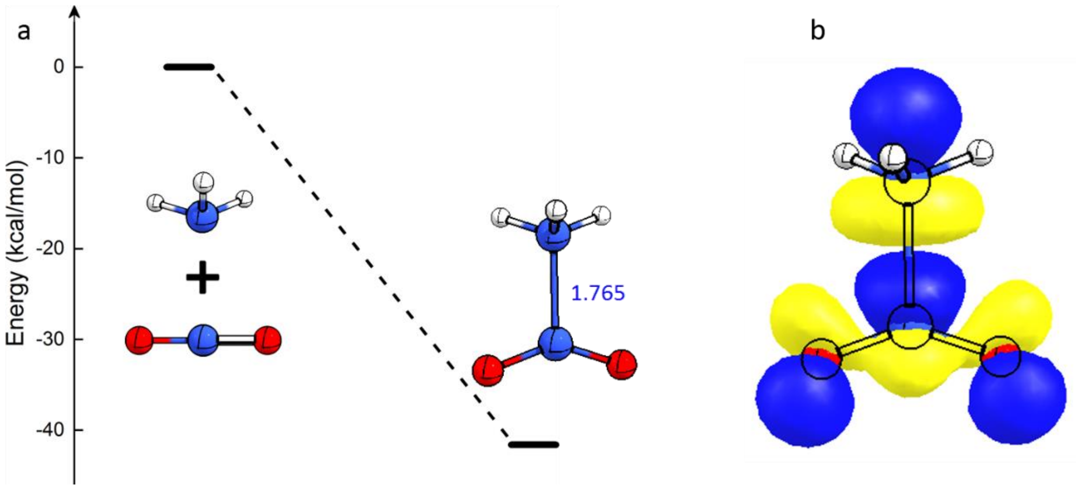

Supplementary Fig. 13. Stability of the intermediate $NH_3NO_2^+$. **a**, Comparison of the energy difference between the intermediate $H_3NNO_2^+$, and the isolated NO_2^+ ion and NH_3 molecule. The white, red and blue spheres represent hydrogen (H), oxygen (O), and nitrogen (N) atoms, respectively. **b**, Orbital for the N-N bond formation in the intermediate $H_3NNO_2^+$

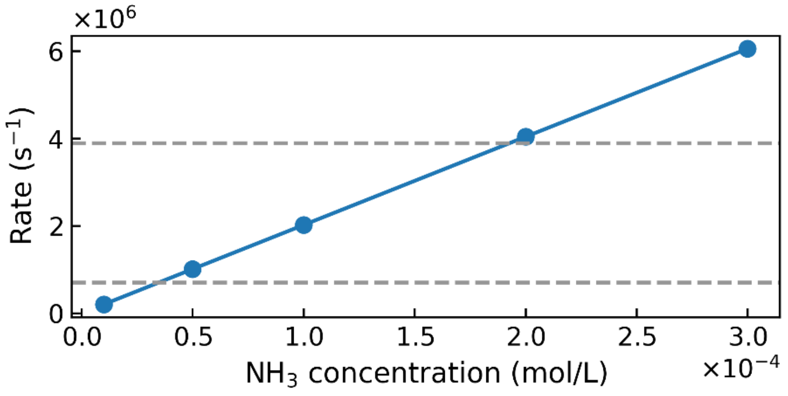

Supplementary Fig. 14. Variation of ammonolysis rate with ammonia concentration. Reaction rate of ammonolysis as a function of NH_3 concentration. Upper and lower hydrolysis rate of N_2O_5 in liquid water are represented by dashed line, obtained by setting the transmission coefficient equal to the upper (i.e., k = 0.5) and lower (i.e., k = 0.1) extremes, respectively.

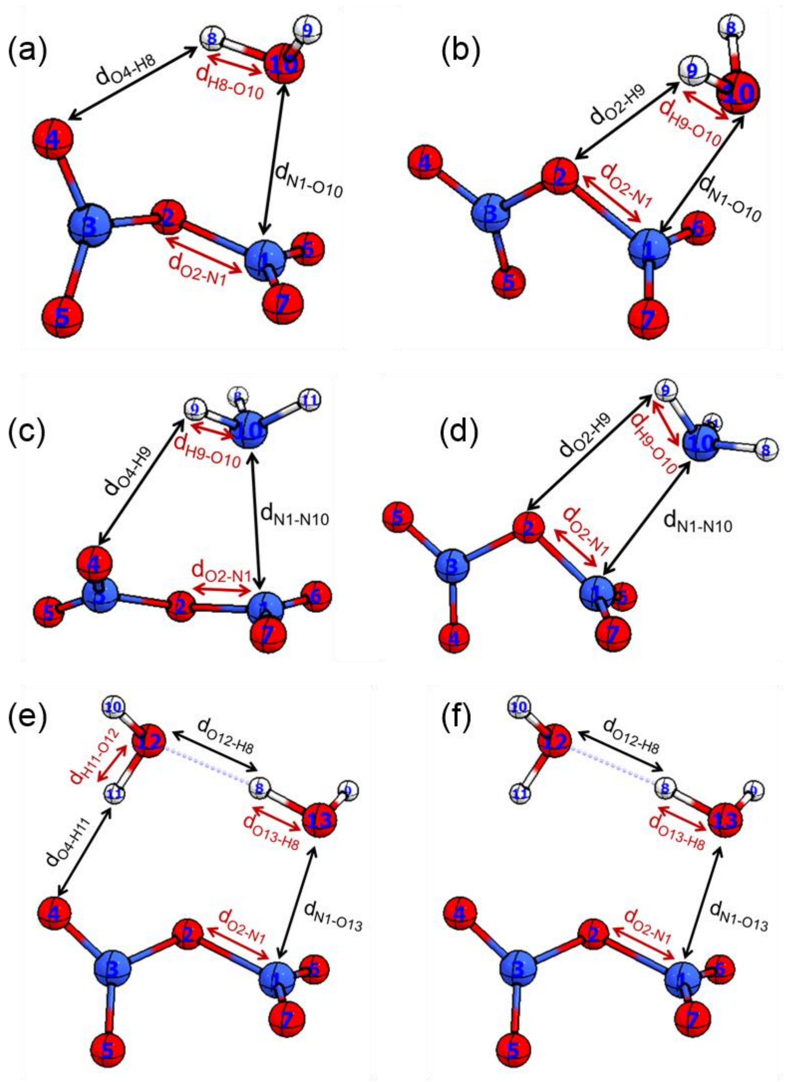

Supplementary Fig. 15. Collective variables (CV) defined for N_2O_5 hydrolysis and ammonolysis. The chosen CVs were linear combinations of the distances of the chemical bonds formed or broken during the reaction observed in MetaD-biased QM/MM simulations, which are defined as follows:

For reaction pathway in (a): $d_{CV} = d_{H8\text{-}O10} + d_{O2\text{-}N1} - d_{O4\text{-}H8} - d_{N1\text{-}O10}$;

For reaction pathway in (b): $d_{CV} = d_{H9\text{-}O10} + d_{O2\text{-}N1} - d_{O2\text{-}H9} - d_{N1\text{-}O10}$;

For reaction pathway in (c): $d_{CV} = d_{H9\text{-}O10} + d_{O2\text{-}N1} - d_{O4\text{-}H9} - d_{N1\text{-}N10}$;

For reaction pathway in (d): $d_{CV} = d_{H9\text{-}O10} + d_{O2\text{-}N1} - d_{O2\text{-}H9} - d_{N1\text{-}N10}$;

For reaction pathway in (e): $d_{CV} = d_{H11\text{-}O12} + d_{H8\text{-}O13} + d_{O2\text{-}N1} - d_{O4\text{-}H11} - d_{O12\text{-}H8} - d_{O13\text{-}N1}$;

For reaction pathway in (f): $d_{CV} = d_{H8\text{-}O13} + d_{O2\text{-}N1} - d_{O12\text{-}H8} - d_{O13\text{-}N1}$.

The white, red and blue spheres represent hydrogen (H), oxygen (O), and nitrogen (N) atoms, respectively.

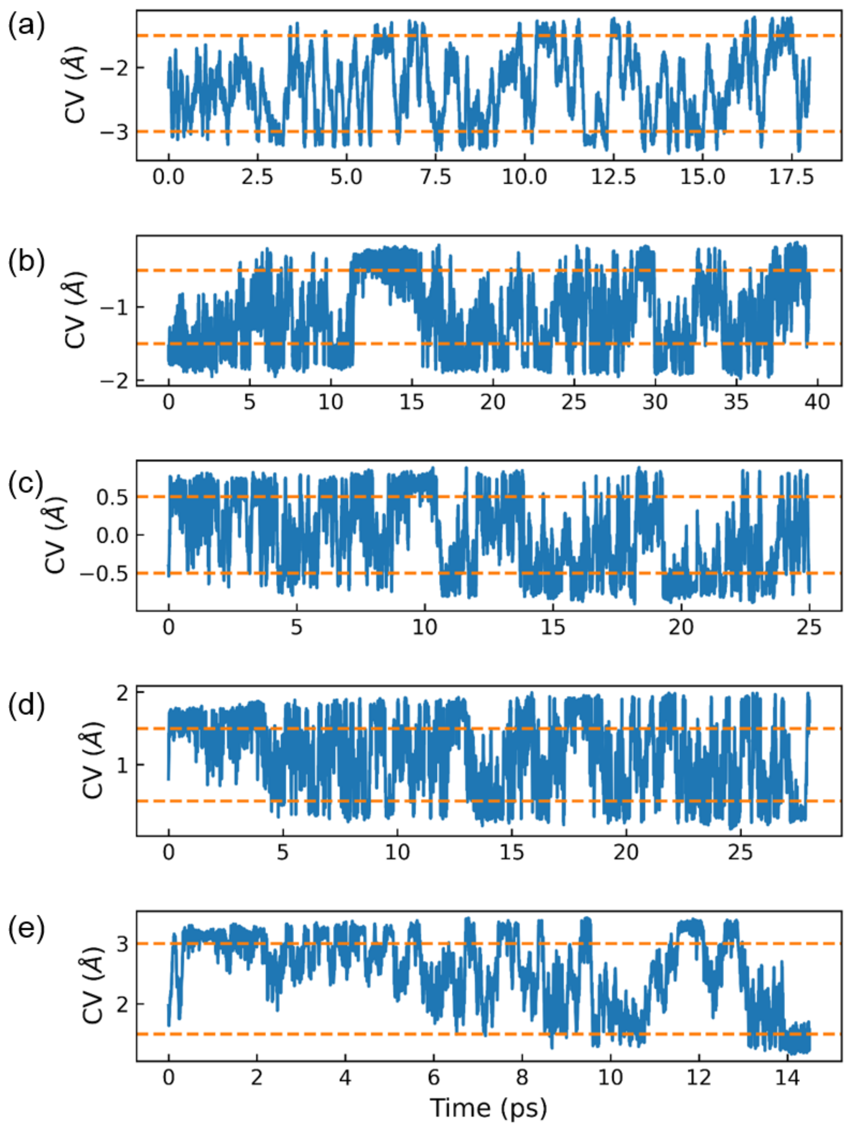

Supplementary Fig. 16. Evaluate the convergence of SMS-MetaD simulations by CV diffusion. Time evolution of the CV in five windows (a-e) of the simulations of the N_2O_5 hydrolysis in bulk water via the ionic mechanism using the SMS-MetaD approach. The orange dashed lines indicate the upper and lower walls applied. The free diffusion of the CV indicates that the convergence has been reached.

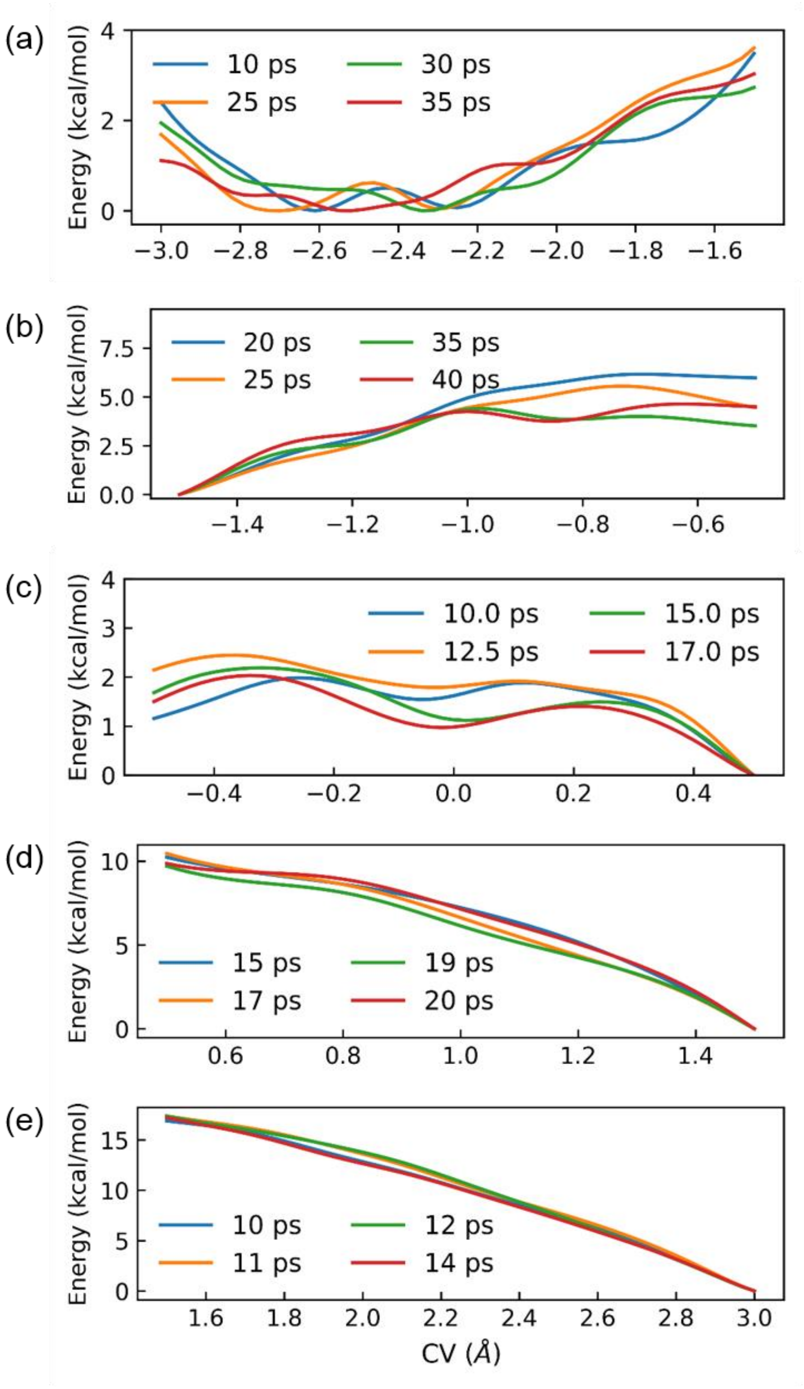

Supplementary Fig. 17. Evaluate the convergence of SMS-MetaD simulations by the variation of free energy with time. Calculated free energy as a function of the selected CV at different times in five windows (a-e) of the simulations of the N_2O_5 hydrolysis in bulk water via the ionic mechanism using the SMS-MetaD approach. The free energy profiles obtained at different times in last several ps do not show significant changes, indicating that convergence has been reached.

Reference:

1. Galib M, Limmer DT. Reactive uptake of N_2O_5 by atmospheric aerosol is dominated by interfacial processes. *Science* **371**, 921-+ (2021).
2. Cruzeiro VWD, Galib M, Limmer DT, Gotz AW. Uptake of N_2O_5 by aqueous aerosol unveiled using chemically accurate many-body potentials. *Nat Commun* **13**, (2022).
