# Supplementary material for: Mechanistic insight into the competition between interfacial and bulk reactions in microdroplets through N2O5 ammonolysis and hydrolysis
Source: Nat Commun. 2024 Mar 15;15:2347. doi: 10.1038/s41467-024-46674-1 (PMC10943240; doi:10.1038/s41467-024-46674-1)
Supplement: Supplementary file 3 — Description of Additional Supplementary Files [file 41467_2024_46674_MOESM3_ESM.pdf]

Description of Additional Supplementary Files

File name: Supplementary Movie 1

Description: A metadynamics-biased QM/MM MD simulation of the reaction of N_2O_5 with water monomer via molecular mechanism.The H group of H_2O attach to the terminal oxygen atom of O_2NONO_2.

File name: Supplementary Movie 2

Description: A metadynamics-biased QM/MM MD simulation of the reaction of N_2O_5 with water monomer via molecular mechanism.The H group of H_2O attach to the central oxygen atom of O_2NONO_2.

File name: Supplementary Movie 3

Description: A metadynamics-biased QM/MM MD simulation of the hydrolysis of N_2O_5 via ionic mechanism.

File name: Supplementary Movie 4

Description: A metadynamics-biased QM/MM MD simulation of the hydrolysis of N_2O_5 via stepwise ionic mechanism.

File name: Supplementary Movie 5

Description: A metadynamics-biased QM/MM MD simulation of the reaction of N_2O_5 with water dimer via molecular mechanism.

File name: Supplementary Movie 6

Description: A metadynamics-biased QM/MM MD simulation of the reaction of N_2O_5 with water trimer via molecular mechanism.

File name: Supplementary Movie 7

Description: A unbiased QM/MM MD simulation of the ammonolysis of N_2O_5 at the air-water interface via molecular mechanism.

File name: Supplementary Movie 8

Description: A unbiased QM/MM MD simulation of the ammonolysis of N_2O_5 at the air-water interface via stepwise ionic mechanism.
